# Fluorescence-Based Bioassays for the Detection and Evaluation of Food Materials

**DOI:** 10.3390/s151025831

**Published:** 2015-10-13

**Authors:** Kentaro Nishi, Shin-Ichiro Isobe, Yun Zhu, Ryoiti Kiyama

**Affiliations:** 1 Department of Applied Chemistry and Biochemistry, Faculty of Engineering, Kyushu Sangyo University, 2-3-1 Matsukadai, Kasi-i, Higashi-ku, Fukuoka 813-8503, Japan; E-Mails: nishi@ip.kyusan-u.ac.jp (K.N.); isobe@ip.kyusan-u.ac.jp (S.I.); 2 Biomedical Research Institute, National Institute of Advanced Industrial Science and Technology (AIST), 1-1-1 Higashi, Tsukuba, Ibaraki 305-8566, Japan; E-Mail: zhu-y@aist.go.jp; 3 Scinet Company, 4-21-12 Takanawa, Minato-ku, Tokyo 108-0074, Japan

**Keywords:** fluorescent dye, bioassay, food study, microarray, signaling pathway

## Abstract

We summarize here the recent progress in fluorescence-based bioassays for the detection and evaluation of food materials by focusing on fluorescent dyes used in bioassays and applications of these assays for food safety, quality and efficacy. Fluorescent dyes have been used in various bioassays, such as biosensing, cell assay, energy transfer-based assay, probing, protein/immunological assay and microarray/biochip assay. Among the arrays used in microarray/biochip assay, fluorescence-based microarrays/biochips, such as antibody/protein microarrays, bead/suspension arrays, capillary/sensor arrays, DNA microarrays/polymerase chain reaction (PCR)-based arrays, glycan/lectin arrays, immunoassay/enzyme-linked immunosorbent assay (ELISA)-based arrays, microfluidic chips and tissue arrays, have been developed and used for the assessment of allergy/poisoning/toxicity, contamination and efficacy/mechanism, and quality control/safety. DNA microarray assays have been used widely for food safety and quality as well as searches for active components. DNA microarray-based gene expression profiling may be useful for such purposes due to its advantages in the evaluation of pathway-based intracellular signaling in response to food materials.

## 1. Introduction

Fluorescent dyes or fluorophores have been widely used as probes (for physical and structural parameters), indicators (e.g., for molecular concentrations) or labels/tracers (e.g., for visualization and localization of biomolecules) in various bioassays [1]. While the development of fluorescent dyes has a history many centuries long, their importance has increased due to the recent advancement of new fluorescent dyes [2], which have been developed along with the development of new biotechnological tools and devices. For example, Laurdan, a naphthalene-based amphiphilic fluorescent dye having as characteristics the ability to penetrate membranes and a large Stokes shift, was developed to study membrane fluidity and dynamics, and its usage was made quite effective by the development of two-photon fluorescent microscopy, a microscope system with two-photon excitation, which enables the detection of signals with less background, less photodamage and more depth discrimination [3,4,5].

Therefore, the development of fluorescent dyes has had quite an impact when accompanied by the development of suitable devices and their applications. One of the most important currently emerging research fields is the development and application of technologies for new functional foods and quality control and safety of its production. Along with technological innovations, the effective usage of gene/genome information in the pathway-based evaluation of materials is crucial. We summarize here recent progress in fluorescence-based bioassays, including genomic and transcriptomic assays, by focusing on their applications in the study of food safety, quality and efficacy.

### 1.1. Overview of Fluorescent Dyes

Fluorescent dyes are generally polyaromatic or heterocyclic hydrocarbons, which undergo a three-stage process of fluorescence: excitation, excited-state lifetime and fluorescence emission [6]. Fluorescent dyes are characterized by key properties, such as those revealed by the absorption maximum (λ_max_), the emission maximum (λ_em_), the extinction coefficient (ε) and the fluorescence quantum yield (Φ) [2]. For example, the “Stokes shift”, defined by the difference between λ_max_ and λ_em_, is an important property of a fluorescent dye, and a large Stokes shift helps to avoid the reabsorption of emitted photons, giving higher contrast in fluorescent imaging [7].

New technologies, materials and devices have been developed for the efficient detection and utilization of the fluorescence signals in a biological specimen. For example, fluorescence-activated cell sorting (FACS) is an example of the successful application of fluorescence technologies for flow cytometry, and is now used in basic as well as industrial fields of life science [8,9]. Flow cytometry is a technique used for cell counting, cell sorting and biomarker detection, by passing a cell suspension in a stream of fluid through an electronic detection apparatus, allowing simultaneous multiparametric analyses of many thousands of micrometer-sized particles per second. Its applications include food study, such as water testing, milk analysis, brewing/wine production and food microbiology [10]. Meanwhile, fluorescence *in situ* hybridization (FISH) is a cytogenetic technique in which fluorescently labeled probes are hybridized with parts of DNA on chromosomes or specific RNA targets (e.g., mRNA and miRNA), and signals are detected by fluorescence microscopy. After a 30-year history, the original FISH protocol has been diversified into a number of new protocols with improved sensitivity, specificity and resolution [11]. For example, chromosome orientation-FISH, or CO-FISH, can detect strand-specific target DNA, and thus is useful to detect chromosomal abnormalities, such as Robertsonian translocations, chromosomal inversion and telomeric alterations [12].

A number of fluorescent techniques utilize Förster resonance energy transfer (FRET), a mechanism of energy transfer from a donor dye to a different acceptor dye, which is used to analyze conformations, interactions and concentrations of proteins and nucleic acids [6]. Protein-protein interactions can be detected by other fluorescent techniques, such as bioluminescence resonance energy transfer (BRET) assay, a modification of FRET, and biomolecular fluorescence complementation (BiFC) assay. BiFC assay is based on structural complementation between two non-fluorescent N- and C-terminal fragments of a fluorescent protein, and has contrasting advantages and disadvantages compared with FRET [13,14].

Other than aromatic hydrocarbons, several unique materials have also been utilized for fluorescence applications. Quantum dots are fluorescent semiconductor nanoparticles that have potential in biology, such as specific labeling of cells and tissues, long-term imaging, lack of cytotoxicity, *in vivo* multicolor imaging and FRET-based sensing [15]. A variety of fluorescent colors are available, depending on the size and shape of the particles. Additionally, some lanthanide ions are useful for bioassays due to their superior characteristics, such as long fluorescent lifetimes, large Stokes shifts and sharp emission profiles [16]. These materials have been used to study food safety, quality and efficacy (see Section 2).

### 1.2. Fluorescent Dyes for Bioassays

Fluorescent probes are required to match certain conditions for experiments, such as wavelength range, Stokes shift and spectral bandwidth, which are partly imposed by the instrumentation and the requirements of multicolor labeling experiments [6]. To design fluorescent experiments, the fluorescent output of a dye judged by the extinction coefficient and the fluorescence quantum yield needs to be considered. Additionally, under high-intensity illumination conditions, the irreversible destruction or photobleaching of fluorescent dyes is an important factor. Polyaromatic fluorescent dyes with extended π-conjugated systems could thus be ideal for designing dyes with longer Stokes shifts [7], which may improve the performance of fluorescent dyes. Here, we summarize the fluorescent dyes frequently used for bioassays.

Since its first synthesis in 1871, fluorescein, along with its derivatives, has been used as a powerful tool in various fields of life science [17]. Fluorescein is composed of two parts of xanthene, the chromophore part, and benzene, and exhibits excitation at 490 nm and emission at 514 nm (λ_max_/λ_em_ = 490/514 nm), with fluorescent properties of ε = 9.3 × 10^4^ M^−1^·cm^−1^ and Φ = 0.95 [2]. A variety of fluorescein derivatives have been synthesized to improve its chemical, fluorescent and biological properties, and its stability, such as Oregon Green, fluorescein isothiocyanate (FITC), fluorescein diacetate and carboxyfluorescein (FAM). These dyes and fluorescein have been used in various bioassays/biomaterials, such as cell assays (flow cytometry, suspension arrays, fluorescent microscopy, fluorescent cell assay and fluorescent cytomics), FRET-based assays, probing (CO-FISH, fluorescent caspase assay, fluorescent hybridization, fluorescent nanoparticle assay, fluorescent nucleic acid assay and small-molecule fluorochrome assay) and microarray/biochip assays (see Section 2.1).

Rhodamines are isologs of fluorescein, having two amino groups, one of which is positively charged, and have properties similar to fluorescein, such as λ_max_/λ_em_ = 496/517 nm, ε = 7.4 × 10^4^ M^−1^·cm^−1^ and Φ = 0.92 for rhodamine 110 [2]. Rhodamine derivatives were developed for imaging, such as carboxytetramethylrhodamine (TAMRA), tetramethylrhodamine (TMR) and its derivative (tetramethylrhodamine isothiocyanate or TRITC), or to improve photostability and increase brightness, such as Alexa Fluor and DyLight Fluor dyes. Rhodamines (rhodamine, rhodamine B, lissamine rhodamine B, sulforhodamine B, Texas Red, TMR and TRITC) were extensively used in various bioassays/biomaterials, such as cell assays (fluorescent cytomics), probing (fluorescent hybridization, fluorescent nanoparticle assay, fluorescent nucleic acid assay and small-molecule fluorochrome assay) and microarray/biochip assays (see Section 2.1).

Cyanines are composed of two quaternized heteroaromatic bases joined by a polymethine chain, and their colors depend on the number of carbons (3 for Cy3 and 5 for Cy5) in the polymethine chain. Among cyanines, Cy3 and Cy5 have been most utilized, and while Cy3 shows fluorescent properties of λ_max_/λ_em_ = 554/568 nm, ε = 1.3 × 10^5^ M^−1^·cm^−1^ and Φ = 0.14, Cy5 shows those of λ_max_/λ_em_ = 652/672 nm, ε = 2.0 × 10^5^ M^−1^·cm^−1^ and Φ = 0.18 [2]. Cy3 and Cy5 have been used cooperatively and/or complementarily in multi-parameter fluorescence imaging [18], or as test/reference microarray probes [19] or photoconvertible fluorescent probes [20]. Cyanines have been used in various bioassays/biomaterials, such as probing (CO-FISH, fluorescent nanoparticle assay, fluorescent nucleic acid assay, fluorescent spectroscopy and FRET-based assays), protein/immunological assays (sandwich fluoroimmunoassay) and microarray/biochip assays (see Section 2.1).

Alexa Fluor dyes are synthesized through the sulfonation of coumarin, rhodamine, xanthene and cyanine dyes, and have characteristics of greater photostability and brightness as well as lower pH sensitivity than common dyes with comparable excitation/emission [21]. Among Alexa Fluor dyes, Alexa Fluor 488 (green; λ_max_/λ_em_ = 495/519 nm, ε = 7.3 × 10^4^ M^−1^·cm^−1^ and Φ = 0.92), Alexa Fluor 546 (orange; λ_max_/λ_em_ = 556/573 nm, ε = 1.1 × 10^5^ M^−1^·cm^−1^ and Φ = 0.79), Alexa Fluor 555 (red-orange; λ_max_/λ_em_ = 555/565 nm, ε = 1.6 × 10^5^ M^−1^·cm^−1^ and Φ = 0.10) and Alexa Fluor 647 (far-red; λ_max_/λ_em_ = 650/668 nm, ε = 2.7 × 10^5^ M^−1^·cm^−1^ and Φ = 0.33) were frequently used in bioassays [6]. Alexa Fluor dyes have been used in various bioassays/biomaterials, such as biosensing (magnetic modulation biosensing), probing (small-molecule fluorochrome assay) and microarray/biochip assays (see Section 2.1).

Green fluorescent protein (GFP) of the jellyfish Aequorea victoria is a protein composed of 238 amino acid residues, which has an eleven-stranded β barrel with an α helix covalently bonded with a chromophore running through the center [22]. GFP has two excitation peaks, at 395 (major) and 475 (minor) nm, an emission peak at 508 nm and fluorescent quantum yield of 0.77 [23]. To improve brightness, longer wavelengths and FRET, several mutant GFPs were developed, which include blue fluorescent protein (BFP), cyan fluorescent protein (CFP) and yellow fluorescent protein (YFP) [23,24]. Fluorescent proteins have been used in various bioassays/biomaterials, such as biosensing (fluorescent molecular biosensing), cell assays (flow cytometry, suspension arrays, fluorescent microscopy, fluorescent cell assay, fluorescent reporter-gene assay and single live-cell imaging), FRET-based assays, probing (fluorescent caspase assay and fluorescent reporter assay), protein/immunological assays (BiFC) and microarray/biochip assays (see Section 2.1).

Fluolid dyes are organic electroluminescence dyes, which were developed to overcome the inconvenience of currently available fluorescent reagents, and thus have larger Stokes shifts (more than 120 nm), greater photostability (stable for more than 10 years at room temperature) and more fluorescence in a solid state [25]. Their fluorescent properties are as follows: Fluolid-Green (λ_max_/λ_em_ = 395/522 nm), Fluolid-Yellow (λ_max_/λ_em_ = 410/541 nm), Fluolid-Orange (λ_max_/λ_em_ = 440/602 nm) and Fluolid-Red (λ_max_/λ_em_ = 525/660 nm). Owing to their extraordinary stability, Fluolid dyes have been used with a fluorescence scanning electron microscope (FL-SEM) [25] as well as in DNA microarray assay [26] and immunohistochemistry [27].

Fluorescent dyes and proteins other than those described above have also been used in bioassays, which include DAPI (λ_max_/λ_em_ = 350/450 nm, ε = 1.2 × 10^5^ M^−1^·cm^−1^ and Φ = 0.83) [28], SYBR Green I (λ_max_/λ_em_ = 497/520 nm and Φ = 0.8) [6] and RiboGreen (λ_max_/λ_em_ = 500/525 nm, ε = 6.7 × 10^4^ M^−1^·cm^−1^ and Φ = 0.65) [29] for staining DNA or RNA; R-phycoerythrin (PE: λ_max_/λ_em_ = 546/578 nm, ε = 2.0 × 10^6^ M^−1^·cm^−1^ and Φ = 0.98) [28] for immunofluorescence assays; Texas Red (TxR: λ_max_/λ_em_ = 596/620 nm, ε = 8.5 × 10^4^ M^−1^·cm^−1^ and Φ = 0.51) [28,30] for immunohistochemistry; and NanoOrange (λ_max_/λ_em_ = 582/605 nm and Φ = 0.36 in the protein complex) [31] for protein quantification. These dyes have been used in various bioassays/biomaterials for food study, such as cell assays (flow cytometry and suspension arrays), FRET-based assays, probing (CO-FISH), protein/immunological assays (fluorescent protein assay, fluorescent amplification catalyzed by T7 polymerase technique or FACTT, and real-time immune-PCR) and microarray/biochip assays (see Section 2.1).

### 1.3. Fluorescent Dyes Used in DNA Microarray Assay

Fluorescent dyes play important roles in DNA microarray assays due to their detectivity, speed and increased safety [28]. Fluorescent dyes frequently used in DNA microarray assays are phycoerythrin, Alexa Fluor dyes and cyanines. They have been used either as a single dye, such as phycoerythrin, or as two-color fluorescent probes, such as Cy3/Cy5 and Alexa Fluor 555/647. Owing to their superior reliability, Cy3 and Cy5 have been frequently used in gene expression profiling by means of DNA microarray assays since the early days [19]. Cyanines are, however, suggested to have low photostability and to be destabilized by their negative charges [32], as well as being affected by atmospheric ozone in the laboratory [33] and fluorescence quenching [34]. Alexa Fluor dyes, on the other hand, show greater brightness and photostability than cyanines [6]. Phycoerythrin is used in Affymetrix GeneChip assay as a streptavidin-conjugated form to detect biotinylated target cRNA hybridized with the probes on the platform. However, a significant decrease in fluorescent intensity was observed for phycoerythrin [35]. Mitsubishi Rayon developed a hollow fiber array, Genopal, in which fibers are filled with hydrogels attached to oligonucleotide probes and Cy5-labeled target cDNA is hybridized with the probes [36]. Although cyanines were generally used to label probes in the DNA microarray assay developed by GE Healthcare, a fluorescent dye, Amersham HyPer5, was developed and used to label target DNA [37]. Fluorescence-based microarray/biochip assays are summarized below (see Section 2.3 and Section 3.1).

## 2. Application of Fluorescence-Based Bioassays

Fluorescence-based bioassays have been applied in biotechnology and various fields in life science. For example, various fluorescently labeled antibodies have been used to detect specific organelles, cellular activities (e.g., cell morphology, viability and functions) and cellular processes (e.g., transportation, endocytosis and receptor function) [38]. Clinical and pharmacological applications of fluorescent probes have been explored to diagnose leukemia and other cancers [39]. These applications are supported by basic characteristics of fluorophores, such as structural and environmental effects on fluorescence emission, fluorescence polarization and FRET, which are applied for spectrofluorometry, fluorescent microscopy and fluorescence-based chemical sensing to trace and image biological objects [1]. In this section, we summarize first fluorescence-based bioassays and then their applications by discussing representative literature.

### 2.1. Fluorescence-Based Bioassays

Fluorescence-based bioassays, classified into biosensing, cell assays, energy transfer-based assays, probing, protein/immunological assays and microarray/biochip assays, are summarized in Table 1. Biosensing, such as fluorescent molecular biosensor, fluorometric high-performance liquid chromatography (HPLC) and magnetic modulation biosensing, has been used to detect intermolecular interactions and targets at low concentrations, or to analyze nitrite/nitrate, where fluorescent dyes, such as Alexa Fluor 488, GFP and 2,3-naphthotriazole, have been used. Cell assays, such as flow cytometry (fluorescence-activated cell sorting: FACS), fluorescent cytomics, fluorescence microscopy, fluorescent reporter-gene assay and live-cell imaging, have been used in particle-based flow cytometric assay, drug delivery research and applications of RNA/DNA aptamers to measure cell fluorescence, to screen hormonally active compounds and to examine gene expression/protein interaction, where fluorescent dyes, such as fluoresceins (including FAM and FITC), GFP/GFP-family proteins, lanthanides, phycoerythrin and rhodamines (TMR-C5), have been used. Meanwhile, energy transfer-based assays have been used in live-cell imaging and to analyze protein structure, where fluorescent dyes, such as BFP/GFP, FITC and phycoerythrin, have been used.

A number of technologies have been developed for various types of probe, such as fluorescent calcium indicators, fluorescent caspase substrates, fluorescent nanoparticles, fluorescent nucleic acids, quantum dots and small-molecule fluorochromes, which have been used in fluorescent hybridization (e.g., FISH), reporter-gene assay and fluorescent spectroscopy, often used in combination with FRET, to examine chromosome aberrations/segregations, gene-gene/DNA-protein interactions, calcium signaling and ion channeling; to evaluate fluorescence bioassays, imaging/labeling/sensing, immunoassays/microarray assays, quantitative structure-activity relationship (QSAR), antimycobacterial susceptibility, biological enzymatic reactions, G-protein-coupled receptor (GPCR) ligands and reactive oxygen species; and to screen bladder and other tumor markers, antagonists of GPCRs and anticancer drugs, by using fluorescent dyes, such as Alexa Fluor 546, Cy3/Cy5, FDA, FITC, Fluo-4, fluorescein, FuraRed, GFP/RFP, hydroethidine/hydrocyanines, quantum dots, rhodamine, SpectrumGold/SpectrumOrange, TRITC and Texas Red.

**Table 1:** Fluorescence-based bioassays. BEBO: 4-[(3-methyl-6-(benzothiazol-2-yl)-2,3-dihydro-(benzo-1,3-thiazole)-2-methylidene)]-1-methyl-pyridinium iodide; BFP: blue fluorescent protein; BiFC: bimolecular fluorescent complementation; ELISA: enzyme-linked immunosorbent assay; FACS: fluorescence-activated cell sorting; FACTT: fluorescent amplification catalyzed by T7 polymerase technique; FAM: carboxyfluorescein; FDA: fluorescein diacetate; FISH: fluorescent *in situ* hybridization; FITC: fluorescein isothiocyanate; FRET: fluorescence resonance energy transfer; GFP: green fluorescent protein; GPCR: G-protein-coupled receptor; PCR: polymerase chain reaction; QSAR: quantitative structure activity relationship; RFP: red fluorescent protein; TMR: tetramethylrhodamine; TRITC: tetramethylrhodamine isothiocyanate; TxR: Texas Red; YFP: yellow fluorescent protein.

Bioassay/Biomaterial	Purpose/Subject	Fluorescent Dye/Molecule (Representative)	Reference
**Biosensing**			
Fluorescent molecular biosensing	Detection of intermolecular interactions	GFP	Altschuh *et al.*, 2006 [40]
Fluorometric HPLC	Analysis of nitrite/nitrate	2,3-Naphthotriazole	Jobgen *et al.*, 2007 [41]
Magnetic modulation biosensing	Detection of targets at low concentrations	Alexa Fluor 488	Danielli *et al.*, 2010 [42]
**Cell Assay**			
Flow cytometry/FACS	Particle-based flow cytometric assay	GFP	Vignali, 2000 [43]
Flow cytometry/Suspension array	Measurement of cell fluorescence	GFP/FITC/Phycoerythrin	Edwards *et al.*, 2004 [44]
Fluorescence microscopy	Drug delivery research	GFP/Fluorescein	White & Errington, 2005 [45]
Fluorescent cell assay	High-throughput drug discovery	GFP-family proteins	Wolff *et al.*, 2008 [46]
Fluorescent cell assay	Application in cellular assays	Lanthanides/GFP/FAM	Hanson & Hanson, 2008 [47]
Fluorescent cytomics	Application of RNA/DNA aptamers	Fluorescein/TMR-C5	Ulrich *et al.*, 2004 [48]
Fluorescent reporter-gene assay	Screening of hormonally active compounds	GFP	Svobodová & Cajthaml, 2010 [49]
Single live-cell imaging	Gene expression/Protein interaction	GFP	Mullassery *et al.*, 2008 [50]
**Energy Transfer-Based Assay**			
FRET	Live-cell imaging	GFP/BFP	Salipalli *et al.*, 2014 [51]
FRET/Flow cytometry	Analysis of protein structure	FITC/Phycoerythrin/GFP	Szöllosi *et al.*, 1998 [52]
**Probing**			
FISH	Monitoring of chromosome aberrations	(Not shown)	Léonard *et al.*, 2005 [53]
FISH	Screening of bladder tumor markers	SpectrumGold, *etc.*	Lokeshwar & Selzer, 2006 [54]
FISH	Study of gene-gene/protein interactions	SpectrumOrange, *etc.*	Chun *et al.*, 2009 [55]
FISH (CO-FISH)	Chromosome segregation study	Cy3/Cy5/FITC/TxR	Falconer & Lansdorp, 2013 [56]
Fluorescent calcium indicator	Calcium signaling for cell functions	Fluo-4	Apáti *et al.*, 2012 [57]
Fluorescent caspase substrate/FRET	Screening of anticancer drugs	FITC/GFP/RFP	Brunelle & Zhang, 2011 [58]
Fluorescent hybridization	Identification of nucleic acids	Fluorescein/Rhodamine, *etc.*	Marras *et al.*, 2005 [59]
Fluorescent nanoparticle	Synthesis of fluorescent probes	Cyanines/FITC/TRITC	Sokolova & Epple, 2011 [60]
Fluorescent nucleic acid probe	Labeling of nucleic acid probes	Fluorescein/Rhodamine, *etc.*	Kricka & Fortina, 2009 [61]
Fluorescent reporter assay	Functional study of ion channels	GFP/RFP	Musa-Aziz *et al.*, 2010 [62]
Fluorescent reporter assay/FRET	Antimycobacterial susceptibility testing	FDA/GFP/RFP	Sánchez & Kouznetsov, 2010 [63]
Fluorescent reporter assay/FRET	Detection of gene expression	GFP/RFP	Jiang *et al.*, 2008 [64]
Fluorescent spectroscopy/FRET	Probing biological enzymatic reactions	Cy3/Cy5	Jahnz & Schwille, 2004 [65]
Quantum dot	Fluorescence bioassay	Quantum dot	Liu *et al.*, 2005 [66]
Quantum dot/FRET	Imaging/labeling/sensing	Quantum dot	Medintz *et al.*, 2005 [67]
Quantum dot/FRET	Immunoassay/microarray assay/imaging	Quantum dot	Zhang & Wang, 2012 [68]
Quantum dot/Suspension array	Detection of cancer markers/tumor cells	Quantum dot	Akinfieva *et al.*, 2013 [69]
Small-molecule fluorochrome	Screening of antagonists for GPCRs	FITC/FuraRed/Alexa Fluor 546	Arterburn *et al.*, 2009 [70]
Small-molecule fluorochrome	Detection of reactive oxygen species	Hydroethidine/Hydrocyanines	Maghzal *et al.*, 2012 [71]
Small-molecule fluorochrome	QSAR	FDA	Horobin *et al.*, 2013 [72]
Small-molecule fluorochrome	Fluorescently labeled GPCR ligands	Rhodamine B, *etc.*	Vernall *et al.*, 2014 [73]
**Protein/Immunological Assay**			
BiFC	Protein interaction/modification	GFP/YFP	Kerppola, 2009 [74]
BiFC	Protein-protein interaction	GFP/YFP, *etc.*	Miller *et al.*, 2015 [75]
Chemifluorescent ELISA	Monitoring of kinase activity	(Not shown)	Wu *et al.*, 2010 [76]
Fluorescent dye-based protein assay	Quantitation of protein	NanoOrange	Noble & Bailey, 2009 [77]
Immuno-detection (FACTT)	Quantification of rare blood biomarkers	RiboGreen	Freudenberg *et al.*, 2008 [78]
Lanthanide-doped fluorescent assay	Application for bioassay/therapy	Lanthanides	Guo & Sun, 2012 [79]
Lanthanide fluorescent immunoassay	Time-resolved fluorescence bioassay	Eu^3+^/Sm^3+^/Tb^3+^/Dy^3+^	Yuan & Wang, 2005 [16]
Lanthanide fluorescent immunoassay	Prion disease research	Lanthanides	Sakudo *et al.*, 2007 [80]
Real-time immuno-PCR	Diagnoses of viral antigens/pathogens	SYBR Green I/BEBO	Barletta, 2006 [81]
Sandwich fluoroimmunoassay	Detection/identification of toxins	Cy5	Ligler *et al.*, 2003 [82]
**Microarray/Biochip Assay** (see Table 2)			

Protein/immunological assays, such as BiFC, chemifluorescent enzyme-linked immunosorbent assay (ELISA), fluorescent dye-based protein assay, immuno-detection (FACTT; see Table 1), lanthanide-doped fluorescent assay, lanthanide fluorescent immunoassay, real-time immuno-PCR and sandwich fluoroimmunoassay, have been used to examine bioassay/therapy, kinase activity, protein interaction/modification and time-resolved fluorescence bioassay, to screen/identify rare blood biomarkers and some toxins, to study prion diseases and to identify viral antigens/pathogens, for which fluorescent dyes, such as BEBO (a cyanine dye), Cy5, GFP/YFP, lanthanides (e.g., Eu^3+^, Sm^3+^, Tb^3+^ and Dy^3+^), NanoOrange, RiboGreen and SYBR Green I, have been used. Microarray/biochip assays are discussed below (see Section 2.3). GFP has been used quite often as reporter conjugates in cell assay. For example, estrogen activity was detected by a reporter construct of the human ERα gene fused to yeast enhanced GFP (yEGFP), which was used as a rapid yeast bioassay to screen estrogen activity in calf urine [83]. This construct was validated as a bioassay for hormonal substances in feed [84], and concomitantly improved by the combination with mass-spectrometry techniques [85,86] and by the use of various test samples [87,88], and combined with androgen assay [89], to attain to a level of a standardized multi-hormonal bioassay system.

### 2.2. Fluorescence-Based Microarrays/Biochips

#### 2.2.1. Antibody/Protein Microarray

Protein microarray assay is a high-throughput method used to study biochemical activities of proteins, by measuring their binding affinities, specificities and quantities [90]. The array has a support surface, such as a slide glass, a nitrocellulose membrane, a bead and a microtiter plate, to which the captured protein is bound as an array, and probe molecules, typically labeled with a fluorescent dye or conjugated with enzymes for chemiluminescent or colorimetric assays, are added to the array. In a fluorescent assay, the reaction between the fluorescence-labeled probe and the immobilized protein causes the emission of a fluorescent signal at a specific position, which is detected by a laser scanner. There are three types of protein microarray used to study the biochemical activities of proteins: analytical microarrays, functional protein microarrays and reverse-phase protein microarrays [90]. Antibody microarrays belong to the category of analytical microarrays, and sometimes use a sandwich format consisting of capture antibodies (e.g., biotinylated antibodies), analytes (e.g., toxins) and reporter molecules (e.g., avidin-conjugated nanoparticles and fluorophore-conjugated secondary antibodies). They have been used to screen foodborne pathogens such as *Escherichia coli* O157:H7 and *Salmonella* spp. [91], and to detect multiplex toxins, such as toxins contaminating milk, apple cider and blood samples [92].

#### 2.2.2. Bead/Suspension Array

The detection of bacterial/plant toxins [93], mycotoxins [94] and pesticides [95] in food has been carried out by using bead/suspension array technology, in which fluorescent dye-labeled microspheres/beads are often used. Appropriate molecules or receptors, such as DNA (oligonucleotides), and antibodies and other proteins, are attached to the microspheres differently labeled with fluorescent dyes, for example. Beads are readily suspendable in solution and are used for hybridization between receptors and corresponding reactive biomolecules. Bead arrays have advantages over flat arrays in the array preparation (containing millions of particles per milliliter) and density (containing hundreds of thousands of array elements per microliter), enabling multiparameter detection and high-throughput processing [96]. Since the optical property of each bead is known, target biomolecules hybridized/bound to the beads can be easily differentiated, and quantification can be achieved by comparing the relative intensity of targets in a set of beads with that of markers in another set of beads using fluorescence detection apparatuses, such as a flow cytometer.

#### 2.2.3. Capillary/Sensor Array

A sensor array typically consists of a recognition component, a transducer component and an electronic detection system. The recognition component uses biomolecules to interact with the analyte of interest. This interaction is measured by biotransducers, such as an optical transducer, which outputs a measurable signal proportional to the presence of the target analyte in the sample. Meanwhile, biomolecules are separated first by capillary electrophoresis in an array and then detected by appropriate sensors in capillary arrays. There have been cases of the application of capillary/sensor arrays for food analysis, such as detecting pathogens and toxins, and fluorescent substances are commonly used in their detection systems. Recently, researchers have performed successful analyses of food using improved sensor arrays, such as those with dendritic fluorophores [97] and a fluorescent indicator-displacement sensor array using titania as a host material [98].

#### 2.2.4. DNA Microarray/PCR-Based Array

Using DNA microarray technology, multiple genes can be characterized simultaneously in a single assay. It has been used widely for the analysis of gene expression, but it can also be used for the analysis of microbial pathogens for food safety and environmental applications. A DNA microarray involves the immobilization of numerous probes, such as cDNA and oligonucleotide probes, at a high density on a solid matrix, such as glass, to which fluorescence-labeled PCR-amplified target DNA fragments can be hybridized. The signal generated by the bound labeled targets on the microarray allows identification based on the known locations of the probes on the array. Applications of DNA microarray technology for the detection of pathogens contaminating food have been reported (detailed in Section 3).

#### 2.2.5. Glycan/Lectin Array

The use of glycan microarrays, comprising multiple different glycans on a single platform, is a technique for the analysis of glycosylation patterns and the screening of a number of glycan-binding proteins for investigation of their roles in biological systems. Recently, a shotgun glycan microarray prepared from isolated human milk glycans was reported, where viruses, antibodies and glycan-binding proteins including lectins were detected in order to examine the diverse recognition functions of human milk glycans [99]. In addition, a lectin microarray, based on the specific affinity of a lectin to a specific glycan, is another useful platform for glycan analysis. Recently, a bead-based multiplex lectin array was developed, where respective lectins were coupled to differentially fluorescent dye-coated microbeads [100]. These beads were incubated with biotin-labeled glycoproteins in suspension, with visualization using the interaction between biotin and streptavidin-R-phycoerythrin. This microarray was applied for glycosylation profiling of hepatocellular carcinoma-associated immunoglobulin G in a rapid, sensitive and reproducible manner.

#### 2.2.6. Immunoassay/ELISA-Based Array

An immunoassay is a test that relies on the inherent ability of an antibody to recognize and bind to a specific antigen, which might exist in a complex mixture, to measure the presence and/or concentration of the antigen. In life science research, immunoassays are often used in studies of the biological functions of proteins, while, in industry, immunoassays are used in various applications, such as to detect contaminants in food and water and to monitor and assess specific molecules during food processing. In immunoassays, antibodies or antigens are conjugated or coupled with fluorescent dyes, or labeled with other materials, such as biotin and horseradish peroxidase, to produce measurable fluorescent, chemiluminescent or chromogenic signals for detection. One of the most popular immunoassays is ELISA, in which antigens in a sample are first attached to the surface of the platform (e.g., a 96-well microtiter plate), which are then detected with a specific antibody linked to an enzyme (for enzymatic reactions) or a fluorescently labeled secondary antibody. In recent years, fluorogenic labels, such as cyanines and phycoerythrin, have been used in immunoassays to detect mycotoxins for food safety [101,102].

#### 2.2.7. Microfluidic Chip

Microfluidic chips have been used in many biological fields, such as drug screening and the monitoring of food processing. A microfluidic chip is a set of microchannels molded into a material like glass, silicon or polymer. The microchannels are connected together forming a network, which is connected to the outside by transporting inputs and outputs through the chip platform. The surface patterning of bonded or sealed microchannels in a microfluidic chip can be achieved by technologies such as laminar flow and capillarity, photolithography, microplasmas and electrochemical biolithography [103]. Microfluidic chips have advantages over conventional devices, such as that the assay can be performed on a small scale and thus requires less time and smaller amounts of samples and reagents, and can be performed automatically with high reproducibility [104]. Thus, microfluidic chips have been combined with other systems, such as capillary electrophoresis, PCR and flow cytometry. For example, a simple microfluidic chip system combined with a probe-immobilized fluorescent bead assay was developed for the rapid detection of bacteria associated with food poisoning [105]. Meanwhile, a microfluidic chip system combined with a BRET-based biosensor was developed for real-time, continuous detection with superior sensitivity of maltose in water or beer [106].

#### 2.2.8. Tissue Array

The assay using a tissue array is a high-throughput analysis that utilizes hundreds or up to a thousand separate tissue samples on a single platform. Using this method, tissue samples can be rapidly analyzed by histological analyses, such as immunohistochemistry and FISH, in order to screen genetic or protein markers, or to detect tissues infected with pathogenic/toxigenic factors. Since most dyes currently used for microbial fluorescent staining are toxic or carcinogenic, a tissue array system using brilliant blue FCF, which is a food dye and thus has no toxic effects, was developed and applied for microbial cell fluorescence staining of pathogenic/toxigenic and beneficial fungi and bacteria [107].

### 2.3. Application of Fluorescence-Based Microarrays/Biochips for Food Study

Fluorescence-based microarrays/biochips for food study are summarized in Table 2. Antibody/protein microarrays have been applied to detect/screen foodborne pathogens and toxins, where fluorescent dyes, such as Cy3, fluorescein and RuBpy, have been used. Bead/suspension arrays, such as cytometric bead arrays, liquid/magnetic bead arrays and suspension arrays, have been used to detect/quantify mycotoxins, pathogens, genetically modified maize, pesticides and bacterial/plant toxins, where fluorescent dyes, such as Alexa Fluor 532, Cy3, FITC and phycoerythrin, have been used. Capillary/sensor arrays, such as capillary arrays, chemical sensor arrays and fluorescent sensor arrays, have been used to analyze carbohydrates, fresh fruit juices and various food materials, where fluorescent dyes, such as sulforhodamine B, lissamine rhodamine B and synthetic dendritic fluorophores, have been used. DNA microarrays/PCR-based arrays, such as direct RNA hybridization/microarrays, DNA/PNA microarrays, laser microdissection/microarrays, oligonucleotide microarrays, PCR/bead arrays, PCR/microarrays (mutant analysis by PCR and restriction enzyme cleavage or MAPREC assay, and nucleic acid sequence-based amplification implemented microarray analysis or NAIMA; see Table 2) and PCR/single-base extension-tag arrays, have been used to detect mycoplasmas, pathogenic bacteria, grapevine viruses, genetically modified cotton, pathogenic *Vibrio* spp., genetically modified soybean and seafood-borne pathogens, to screen hypoxia-inducible genes and recombinant flavivirus vaccine strains, to examine genotypes of beef/chicken and gene expression profiles of fungi, and to evaluate the authenticity of ginseng drugs, along with fluorescent dyes, such as Alexa Fluor 546/647, Cy3/Cy5, phycoerythrin, PolyAn-Green/PolyAn-Red, AmCyan1, NIR Dye 700/800, Oyster-550 and quantum dots.

Glycan/lectin arrays have been used for functional glycomic analysis or glycosylation profiling, where Alexa Fluor 488, Cy5 and phycoerythrin have been used as fluorescent dyes. Immunoassay/ELISA-based arrays, such as ELISA chips and immunoassay microarrays, or those used in competitive immunoassay, fluoroimmunoassay and sandwich fluoroimmunoassay, have been used to detect/quantify food allergens, mycotoxins, ochratoxin A, pathogens/toxins, staphylococcal enterotoxin B or to assess food safety, where fluorescent dyes, such as Alexa Fluor 647, Cy3/Cy5, fluorescein, FluoSpheres, phycoerythrin and RuBpy, have been used. Microfluidic chips have been used to detect food poisoning bacteria or single-base mismatches, or to monitor food processing, along with fluorescent dyes, such as Alexa Fluor 647, FAM and GFP. Tissue arrays have also been used to stain microbial cells using brilliant blue FCF.

Fluorescence-based microarrays/biochips can be categorized by the number of target chemicals; either the characterization of a single chemical, or the screening of multiple chemicals from a number of samples or mixtures of chemicals. Among the assays shown in Table 2, antibody/protein microarrays, DNA microarrays, glycan/lectin arrays and tissue arrays are advantageous for profiling and analyzing a single chemical due to the ability of multiple probing, while bead/suspension arrays, capillary/sensor arrays and immunoassay/ELISA-based arrays, microfluidic chips and PCR-based arrays are useful for screening because of their high-throughput processing ability.

**Table 2:** Fluorescence-based microarrays/biochips for food study. ELISA: enzyme-linked immunosorbent assay; FAM: carboxyfluorescein; FITC: fluorescein isothiocyanate; GFP: green fluorescent protein; GMO: genetically modified organism; HPLC: high-performance liquid chromatography; MAPK: mitogen-activated protein kinase; MAPREC: mutant analysis by PCR and restriction enzyme cleavage; NAIMA: nucleic acid sequence-based amplification implemented microarray analysis; PNA: peptide nucleic acid; RuBpy: [Ru(bpy)_3_]Cl_2_/Tris(bipyridine)ruthenium(II) chloride.

Method/Tool	Purpose/Subject	Fluorescent Dye/Molecule	Reference
**Antibody/Protein microarray**			
Antibody microarray	Screening of foodborne pathogens	Cy3/Fluorescein	Gehring *et al.*, 2008 [91]
Antibody microarray	Detection of multiplex toxins	Cy3/RuBpy	Lian *et al.*, 2010 [92]
**Bead/Suspension array**			
Aptamer/Suspension array	Detection of mycotoxins	FITC	Sun *et al.*, 2014 [94]
Cytometric bead array	Detection of pathogens	Alexa Fluor 532/Cy3	Stroot *et al.*, 2012 [108]
Liquid bead array	Genetically modified maize	Phycoerythrin	Han *et al.*, 2013 [109]
Magnetic suspension assay	Quantification of bacterial/plant toxins	Phycoerythrin	Pauly *et al.*, 2009 [93]
Microsphere suspension array	Multiplex mycotoxin detection	FITC	Deng *et al.*, 2013 [110]
Suspension array	Detection of pesticides	Phycoerythrin	Wang *et al.*, 2014 [95]
**Capillary/Sensor array**			
Capillary array electrophoresis	Carbohydrate analysis	Sulforhodamine B	Khandurina *et al.*, 2004 [111]
Chemical sensor array	Discrimination of fresh fruit juices	Lissamine rhodamine B	Tan *et al.*, 2014 [98]
Fluorescent sensor array	Electronic tongue for food analysis	Dendritic fluorophores	Niamnont *et al.*, 2010 [97]
**DNA Microarray/PCR-Based Array**			
Direct RNA hybridization/Microarray	Detection of mycoplasmas	Alexa Fluor 647	Kong *et al.*, 2007 [112]
DNA microarray	Authentication of ginseng drugs	Cy5	Zhu *et al.*, 2008 [113]
DNA microarray	Hypoxia-inducible genes	Phycoerythrin	Otsuka *et al.*, 2010 [114]
DNA microarray	Genotyping of beef/chicken	Cy3/Cy5	Reverter *et al.*, 2014 [115]
DNA microarray	Food safety assessment	PolyAn-Green/PolyAn-Red	Brunner *et al.*, 2015 [116]
Laser microdissection/Microarray	Gene expression profiling of fungi	AmCyan1	Tang *et al.*, 2006 [117]
MAPREC assay	Recombinant flavivirus vaccine strain	NIR Dye 700/800	Bidzhieva *et al.*, 2011 [118]
NAIMA	GMO detection	Oyster-550	Morisset *et al.*, 2008 [119]
Oligonucleotide microarray	Detection of pathogenic bacteria	Quantum dot	Huang *et al.*, 2014 [120]
Oligonucleotide microarray	Detection of grapevine viruses	Cy3	Abdullahi *et al.*, 2011 [121]
PCR/Bead array	Detection of genetically modified cotton	Phycoerythrin	Choi, 2011 [122]
PCR/Microarray	Detection of pathogenic *Vibrio* spp.	Alexa Fluor 546	Panicker *et al.*, 2004 [123]
PCR/Single-base extension-tag array	Seafood-borne pathogens	Cy3	Chen *et al.*, 2011 [124]
PNA microarray	Genetically modified soybean	Cy3/Cy5	Germini *et al.*, 2004 [125]
**Glycan/Lectin Array**			
Glycan microarray	Functional glycomic analysis	Alexa Fluor 488/Cy5	Yu *et al.*, 2012 [99]
Lectin array	Glycosylation profiling	Phycoerythrin	Wang *et al.*, 2014 [100]
**Immunoassay/ELISA-Based Array**			
Competitive immunoassay	Detection of ochratoxin A	Cy5	Ngundi *et al.*, 2005 [126]
ELISA chip	Food safety assessment	Fluorescein	Herrmann *et al.*, 2006 [127]
ELISA chip	Staphylococcal enterotoxin B detection	FluoSpheres	Han *et al.*, 2013 [128]
Fluoroimmunoassay	Detection of food allergens	Alexa Fluor 647	Shriver-Lake *et al.*, 2004 [129]
Fluoroimmunoassay	Detection of mycotoxins	Cy5	Ngundi *et al.*, 2006 [130]
Immunoassay microarray	Detection and quantification of toxins	Cy5	Weingart *et al.*, 2012 [131]
Immunoassay microarray	Multiplex mycotoxin detection	Cy3	Hu *et al.*, 2013 [101]
Immunoassay microarray	Detection of mycotoxins	Phycoerythrin	Peters *et al.*, 2014 [102]
Sandwich fluoroimmunoassay	Detection of pathogens/toxins	Cy5	Ngundi & Taitt, 2006 [132]
Sandwich fluoroimmunoassay	Staphylococcal enterotoxin B detection	RuBpy	Zhang *et al.*, 2011 [133]
**Microfluidic Chip**			
Microfluidic chip	Detection of food poisoning bacteria	Alexa Fluor 647	Ikeda *et al.*, 2006 [105]
Microfluidic chip	Detection of single-base mismatches	FAM	Wang *et al.*, 2013 [134]
Microfluidic chip	In-line monitoring of food processing	GFP	Le *et al.*, 2014 [106]
**Tissue Array**			
Microbial cell fluorescence staining	Microbial staining	Brilliant blue FCF	Chau *et al.*, 2011 [107]

## 3. DNA Microarray-Based Assay for Food Study

DNA microarray-based assay for food study has been compared with other technologies. For example, foodborne diseases are a major issue among global public health problems and the development of rapid detection methods is crucial for their prevention and treatment. Law *et al.* summarized rapid methods for the detection of foodborne bacterial pathogens, such as PCR-based methods, PCR-independent methods, DNA microarray assay, biosensor-based assays and immunological methods, and discussed their principles, applications, advantages and limitations [135]. Nucleic acid-based methods generally give high sensitivity, although they require trained personnel and specialized instruments. Biosensor-based assays, on the other hand, can be used without sample pre-enrichment, although they need improvements for on-site detection. Immunological methods, such as ELISA and flow immunoassay, are currently widely used, but have difficulties when interfering molecules are included in the samples. Josefsen *et al.* compared assays for the rapid monitoring of Campylobacter bacteria in poultry production, and real-time PCR is currently closest to a realistic monitoring system, although other methods, such as microarray PCR, miniaturized biosensors, chromatographic techniques and DNA sequencing, could be considered in the future when cost-effective on-site/at-line monitoring capability is achieved [136]. Gui and Patel discussed the merits of DNA testing, such as DNA microarray assay and next-generation sequencing, to detect *Yersinia* and other foodborne pathogens [137]. DNA testing is generally a high-sensitivity and high-throughput assay, allowing the detection of a single molecule in multiple reactions to be performed at once, thus allowing a range of characteristics to be rapidly and simultaneously determined. However, improvements in sample preparation, data analysis and molecular detection techniques are still needed. Lauri and Mariani compared potentials and limitations among four molecular diagnostic methods: PCR, nucleic acid sequence-based amplification (NASBA), oligonucleotide DNA microarray and ligation detection reaction (LDR), in food safety assessment [138]. While DNA microarrays can be used to detect quite a number of DNA species simultaneously, they are expensive and need more time for processing. DNA-based technologies have been used to assess the safety and quality of food, animal feed and environmental samples, by providing traceability to prevent foodborne diseases and markers to monitor genetically modified organisms [139].

### 3.1. DNA Microarray Assay Protocols

Among microarrays and biochips, DNA microarrays have been developed most extensively and some have already been used to diagnose cancer and other diseases or symptoms [140]. While the traditional solid-phase microarrays contain specific DNA probes attached to the surface of glass, plastic or silicon chips, other types have been developed, which include bead, fiber and electric arrays, where DNA is attached on the surface of latex or polystyrene beads (bead arrays) or attached to gels within plastic hollow fibers (fiber arrays), or an electrical current is generated by redox recycling upon target/probe hybridization (electric arrays). While a variety of DNA microarray assays have been developed, they can be classified into two major types: those for genotyping (e.g., for comparative genomic hybridization, identifying mutations and single-nucleotide polymorphisms and chromatin-immunoprecipitation on a chip) or gene expression analyses (e.g., for gene expression profiling, screening expression marker genes and identifying splice variants). Genotyping is used to detect the contamination of microbes in food, to identify pathogenic/toxic microbial strains/subtypes and to examine the authenticity of plants or the presence of genetically modified organisms by using 16S rRNA genes and/or genomic DNA markers specific to the microbes or the plants. Gene expression profiling, on the other hand, has been used to identify contaminated pathogenic/toxic bacterial strains, to detect specific stress responses and to examine the efficacy of food materials or components by examining the expression of pathogenic/toxic genes, stress-responsive genes and disease/metabolism-associated genes.

Fluorescent dyes, such as cyanines (Cy3 and Cy5), fluoresceins (including FITC and FAM) and Alexa Fluor dyes, have been used in DNA microarray assays. New fluorescent dyes, Fluolid dyes, which have characteristics of higher light/temperature resistance and longer Stokes shifts, have been developed and applied for DNA microarray assays [26]. These fluorescent dyes are used to label target DNA either by direct labeling, where fluorescent dyes directly attached to nucleotides (e.g., deoxyuridine 5′-triphosphate or dUTP) are used to label DNA by nick translation or primer extension, or by indirect labeling, where small nucleotides, such as aminoallyl nucleotides, are used to label DNA first, and the primary amino group attached to DNA is subjected to a reaction with the N-hydroxysuccinimide ester group attached to a fluorescent dye. Alternatively, small nucleotides, such as biotinylated or digoxigenin-labeled ones, are used to label DNA first, and the labeled DNA is then detected by secondary molecules, such as fluorescently labeled streptavidin or anti-digoxigenin antibodies, respectively. Biotinylated or digoxigenin-labeled DNA can alternatively be detected by non-fluorescent assays, such as colorimetric and chemiluminescent ones by using chromogens, such as Seramun Green, Silverquant and True Blue, or chemiluminescent substrates, such as chloro-5-substituted adamantyl-1,2-dioxetane phosphate (CSPD) and luminol (see below).

### 3.2. Application of DNA Microarray Assay for Food Study

DNA microarrays used for food study are summarized in Table 3. DNA microarrays have been used to examine the following subjects: allergies such as latex and/or vegetable food allergy; poisoning by microbes, such as *Bacillus cereus*, *Clostridium botulinum*, *Campylobacter* spp., *Clostridium perfringens*, *Escherichia coli*, *Salmonella enterica* and *Staphylococcus aureus*; toxic effects of cadmium, mycotoxins, silver-nanoparticles and tetrodotoxin; contamination of microbes, such as *Alicyclobacillus* spp., *Arcobacter butzleri*, *Bacillus anthracis*, *Lactobacillus* spp., *Listeria monocytogenes*, *Yersinia enterocolitica* and *Yersinia pestis*; the efficacy of food and food materials, such as that in the absorption of phenolic acids, suppressing cancer, the plasma triglyceride-lowering effect, the lipid consumption in skeletal muscle and the improvement of diabetic symptoms and osteoporosis; mechanisms such as those involved in the response to drought stress, immune stress, inflammation, mucosal IgA antibodies and oxidative stress/DNA damage; and quality control and safety of food, such as the authenticity of food, food safety assessment and the identification of genetically modified organisms.

The food/food materials analyzed by DNA microarray assays include the following: bovine milk, cheese, fish, horseradish, meat (pork and chicken), pancake with chicken, pufferfish, rice and vegetables for the study of allergy, poisoning or toxicity; alfalfa, bread (whole-grain and fiber-rich), cantaloupe, cilantro, egg, fish, juice, maize, meat (beef, pork and poultry meat), milk, mung bean, potato, rice, sausage (Thai Nham) and water for the study of food contamination; beverage, cassava, chitooligosaccharide, dairy, herbs (e.g., licorice and those used in Hochuekkito), high-cholesterol/fat diet, imbibed soybean, phenolic preservatives, pineapple, polyunsaturated fatty acids, psyllium, quercetin, skim milk, sweet corn, tea and xanthan gum for study of their efficacy and mechanisms; and canola, cereal (e.g., barley, oat, rice and wheat), citrinin, cotton, crop, food additives, ginseng, *Kothala himbutu* (a medicinal plant), maize, olive, potato, royal jelly and soybean for the study of food quality control and safety. Other materials besides food are composts, digestates and waste for the study of food contamination.

The types of DNA microarray used can be classified into those for genotyping and gene expression analyses (Table 3). The sources of microarrays are either custom arrays or microarrays supplied by companies, such as Affymetrix (USA), Agilent Technologies (USA), Alere Technologies (Clondiag Chip Technologies, Germany), Amersham/GE Healthcare (USA), GeneSystems (France), Mitsubishi Rayon (Japan) and Pathogen Functional Genomic Research Center (USA).

The types of dye or substrate used to detect the signal are: fluorophors, such as Alexa Fluor 555/647, Cy3/Cy5, fluorescein/6-FAM, phycoerythrin, TAMRA and 3,3′,5,5′,-tetramethylbenzidine (TMB), or chromogens/chemiluminescent or colorimetric substrates for non-fluorescent assays, such as CSPD, luminol, Seramun Green, Silverquant and True Blue.

**Table 3:** Application of DNA microarray assay for detection and evaluation of food materials.

Food Source or Material	Material Detected or Subject Examined	Type of Microarray Used (Source ^a^/Dye)	Reference
**Allergy/Poisoning/Toxicity**			
Bovine milk/Pork	Staphylococcal food poisoning	Genotyping (Clondiag/TMB)	Johler *et al.*, 2011 [141]
Cheese	*Staphylococcus aureus* poisoning	Genotyping (Alere/TMB)	Johler *et al.*, 2015 [142]
Cheese/Fish/Meat, *etc.*	*Staphylococcus aureus* poisoning	Genotyping (Clondiag/TMB)	Baumgartner *et al.*, 2014 [143]
Citrinin	Mycotoxin toxicity	Gene expression (Custom/Cy3, Cy5)	Iwahashi *et al.*, 2007 [144]
Food	*Bacillus cereus* poisoning	Genotyping (Custom, E)	Liu *et al.*, 2007 [145]
Food	Coagulase-negative staphylococci	Genotyping (Custom/Cy5)	Seitter *et al.*, 2011 [146]
Food	69 *Salmonella* virulence genes	Genotyping (Custom/Cy3)	Zou *et al.*, 2011 [147]
Food	*Salmonella* serogroups	Genotyping (Custom, C/SG)	Braun *et al.*, 2012 [148]
Food	*Clostridium perfringens* poisoning	Genotyping (Custom/Cy3, Cy5)	Lahti *et al.*, 2012 [149]
Food	Allergen-specific response	Gene expression (Affymetrix/PE)	Martino *et al.*, 2012 [150]
Food	Staphylococcal food poisoning	Genotyping (Clondiag/TMB)	Wattinger *et al.*, 2012 [151]
Food	Silver-nanoparticle-induced genotoxicity	Gene expression (Agilent/Cy3, Cy5)	Xu *et al.*, 2012 [152]
Food	46 *Salmonella* O serogroups	Genotyping (Custom/Cy3)	Guo *et al.*, 2013 [153]
Food	*Campylobacter* pathotypes	Genotyping (Custom/Cy3)	Marotta *et al.*, 2013 [154]
Food	Botulinum neurotoxin poisoning	Genotyping (Custom/PE)	Vanhomwegen *et al.*, 2013 [155]
Food	117 antibiotic resistance genes	Genotyping (Custom, C/True Blue)	Strauss *et al.*, 2015 [156]
Food additive	Toxicity in liver	Gene expression (Custom/Cy3, Cy5)	Stierum *et al.*, 2008 [157]
Horseradish	Quorum sensing inhibitors	Gene expression (Custom/PE)	Jakobsen *et al.*, 2012 [158]
Meat	Shiga toxin-producing *Escherichia coli*	Genotyping (GeneSystems/6-FAM)	Miko *et al.*, 2014 [159]
Meat	Cephalosporin-resistant *Escherichia coli*	Genotyping (Alere/TMB)	Vogt *et al.*, 2014 [160]
Meat/Milk	Coagulase-negative staphylococci	Genotyping (Custom/Cy3, Cy5)	Even *et al.*, 2010 [161]
Pancake with chicken	*Staphylococcus aureus* poisoning	Genotyping (Clondiag/TMB)	Johler *et al.*, 2013 [162]
Pork	*Salmonella enterica* pathogenicity genes	Genotyping (Custom/Alexa Fluor 555/647)	Hauser *et al.*, 2011 [163]
Pufferfish	Tetrodotoxin accumulation	Gene expression (Custom/Cy3)	Feroudj *et al.*, 2014 [164]
Rice	Cadmium toxicity	Gene expression (Custom, C/CSPD)	Zhang *et al.*, 2012 [165]
Vegetable	Latex and/or vegetable food allergy	Gene expression (Affymetrix/PE)	Saulnier *et al.*, 2014 [166]
**Contamination**			
Alfalfa/Cilantro/Mung bean, *etc.*	Detection of *Yersinia enterocolitica*	Genotyping (Custom/Cy3, Cy5)	Siddique *et al.*, 2009 [167]
Beef	Pathogenic *Escherichia coli* O157	Gene expression (Custom/Cy3, Cy5)	Fratamico *et al.*, 2011 [168]
Beer	Beer spoilage bacterial contamination	Beer spoilage bacterial contamination	Weber *et al.*, 2008 [169]
Beef/Egg/Fish/Milk	26 probes for pathogenic bacteria	Genotyping (Custom/Cy3)	Wang *et al.*, 2007 [170]
Bread (Whole-grain/Fiber-rich)	Intestinal microbiota composition	Genotyping (Agilent/Cy3, Cy5)	Lappi *et al.*, 2013 [171]
Cantaloupe	24 probes for *Listeria monocytogenes*	Genotyping (Affymetrix/PE)	Laksanalamai *et al.*, 2012 [172]
Chicken	Rapid analysis of pathogenic bacteria	Genotyping (Custom/Cy3, Cy5)	Quiñones *et al.*, 2007 [173]
Chicken/Pork	*Salmonella enterica* probes	Genotyping (Custom/Alexa Fluor 555/647)	Hauser *et al.*, 2012 [174]
Compost/Digestate/Waste	Microbial community	Genotyping (Custom/Cy3, Cy5)	Franke-Whittle *et al.*, 2014 [175]
Egg/Meat/Milk, *etc.*	*Listeria* spp. contamination	Genotyping (Custom/Cy5)	Hmaïed *et al.*, 2014 [176]
Egg/Meat/Milk/Rice, *etc.*	16S rRNA probes for pathogens	Genotyping (Custom/Alexa Fluor 647)	Hwang *et al.*, 2012 [177]
Food	250 probes for pathogenic bacteria	Genotyping (Custom/Cy3)	Kim *et al.*, 2008 [178]
Food	Rapid analysis of pathogenic bacteria	Genotyping (Custom/Cy3)	Kim *et al.*, 2010 [179]
Food	Rapid analysis of pathogenic bacteria	Genotyping (Custom, C/Luminol)	Donhauser *et al.*, 2011 [180]
Food	*Yersinia pestis*/*Bacillus anthracis*	Genotyping (Custom/Alexa Fluor 555/647)	Goji *et al.*, 2012 [181]
Food	50 probes for pathogenic bacteria	Genotyping (Custom/Cy3)	Lee *et al.*, 2011 [182]
Food	Diversity of *Arcobacter butzleri*	Genotyping (Custom/Cy3, Cy5)	Merga *et al.*, 2013 [183]
Food	Pathogenic *Escherichia coli*/*Salmonella*	Genotyping (Alere/TMB)	Fischer *et al.*, 2014 [184]
Food/Water	63 probes for pathogenic bacteria	Genotyping (Custom/TAMRA)	Kostić *et al.*, 2010 [185]
Juice	*Alicyclobacillus* spp. contamination	Genotyping (Custom/Cy3, Cy5)	Jang *et al.*, 2011 [186]
Maize	96 probes for mycotoxigenic fungi	Genotyping (Custom/Cy3, Cy5)	Lezar & Barros, 2010 [187]
Meat	Rapid analysis of pathogenic bacteria	Genotyping (Custom/Cy3)	Suo *et al.*, 2010 [188]
Meat (Ready-to-eat)	*Listeria monocytogenes* contamination	Gene expression (PFRGC/Cy3, Cy5)	Bae *et al.*, 2011 [189]
Potato	DNA/RNA pathogens	Genotyping (Custom, C/SG)	Dobnik *et al.*, 2014 [190]
Poultry meat	102 pathogenicity genes	Genotyping (Custom/Alexa Fluor 555/647)	Toboldt *et al.*, 2014 [191]
Sausage (Thai Nham)	164 probes for lactobacilli	Genotyping (Custom/Cy3, Cy5)	Rungrassamee *et al.*, 2012 [192]
Water	26 probes for pathogenic bacteria	Genotyping (Custom/Cy3)	Zhou *et al.*, 2011 [193]
Water	Pathogenic *Legionella* spp.	Genotyping (Custom/Cy3)	Cao *et al.*, 2014 [194]
**Efficacy/Mechanism**			
Beverage/Dairy/Food	Interaction between yeast and bacteria	Gene expression (Affymetrix/PE)	Mendes *et al.*, 2013 [195]
Cassava	Drought stress response	Gene expression (Custom/Cy3)	Utsumi *et al.*, 2012 [196]
Chitooligosaccharide	Immune responses in adipocytes	Gene expression (Illumina/NS)	Choi *et al.*, 2012 [197]
Food	Metabolic change in white blood cells	Gene expression (Affymetrix/PE)	Kawakami *et al.*, 2013 [198]
Food (High-cholesterol diet)	Osteoporosis risk	Gene expression (Affymetrix/PE)	You *et al.*, 2011 [199]
Food (High-fat diet)	Inflammation-associated genes	Gene expression (Illumina/NS)	Ding *et al.*, 2014 [200]
Herb (Hochuekkito)	Mucosal IgA antibody response	Gene expression (Custom/Cy3)	Matsumoto *et al.*, 2010 [201]
Herb (Licorice)	Estrogen-like effect	Gene expression (Custom/ Cy3, Cy5)	Dong *et al.*, 2007 [202]
Imbibed soybean	New protein food item	Gene expression (Custom/Cy3)	Tamura *et al.*, 2014 [203]
Phenolic preservative	Oxidative stress/DNA damage	Gene expression (Custom/Cy3, Cy5)	Martín *et al.*, 2014 [204]
Pineapple (*Ananas comosus*)	Absorption of phenolic acid	Gene expression (Custom/Cy3, Cy5)	Dang & Zhu, 2015 [205]
Polyunsaturated fatty acid, *etc.*	Growth and metabolic status of rats	Gene expression (Illumina/Cy3)	Castañeda-Gutiérrez *et al.*, 2014 [206]
Psyllium	Lipid consumption in skeletal muscle	Gene expression (Mitsubishi/Cy5)	Togawa *et al.*, 2013 [207]
Quercetin	Improvement of diabetic symptoms	Gene expression (Affymetrix/PE)	Kobori *et al.*, 2009 [208]
Skim milk	Survival of *L. monocytogenes*	Gene expression (Custom/Alexa Fluor 555/647)	Liu & Ream, 2008 [209]
Sweet corn	Effect of suppressing cancer	Gene expression (GE Healthcare/Cy5)	Tokuji *et al.*, 2009 [210]
Tea (*Eucommia ulmoides*)	Plasma triglyceride-lowering effect	Gene expression (Agilent/Cy3)	Kobayashi *et al.*, 2012 [211]
Xanthan gum	*Xanthomonas arboricola* metabolism	Genotyping (Custom/Cy3, Cy5)	Mayer *et al.*, 2011 [212]
**Quality Control/Safety**			
Canola	Genetically modified organism	Genotyping (Custom/Cy3)	Schmidt *et al.*, 2008 [213]
Canola/Cotton/Maize/Soybean	Genetically modified organism	Genotyping (Custom/Cy5)	Kim *et al.*, 2010 [214]
Cereal (Barley/Oat/Rice/Wheat)	Authenticity of plant	Genotyping (Custom/Fluorescein)	Rønning *et al.*, 2005 [215]
Crop	Authenticity of food	Genotyping (Custom/Cy3)	Voorhuijzen *et al.*, 2012 [216]
Ginseng	Food adulteration	Genotyping (Custom/Cy3)	Niu *et al.*, 2011 [217]
Kothala himbutu (Medicinal plant)	Food safety assessment	Genotyping (Affymetrix/PE)	Im *et al.*, 2008 [218]
Maize/Potato	Food safety assessment	Gene expression	van Dijk *et al.*, 2010 (review) [219]
Maize/Soybean, *etc.*	Genetically modified organism	Genotyping (Custom, C/Silverquant)	Leimanis *et al.*, 2006 [220]
Olive	Authenticity of plant	Genotyping (Custom/Cy3, Cy5)	Consolandi *et al.*, 2007 [221]
Potato	Food safety assessment	Gene expression (Custom/Cy3, Cy5)	van Dijk *et al.*, 2009 [222]
Royal jelly	Food safety assessment	Gene expression (Amersham/Cy5)	Kamakura *et al.*, 2005 [223]

^a^ The sources of DNA microarrays are either custom arrays (Custom) or microarrays supplied by companies as follows: Agilent: Agilent Technologies, USA; Alere: Alere Technologies, Germany; Amersham/GE Healthcare: GE Healthcare, USA; Clondiag: Clondiag Chip Technologies (renamed as Alere Technologies), Germany; GeneSystems: GeneSystems, France; Mitsubishi: Mitsubishi Rayon, Japan; and PFRGC: Pathogen Functional Genomic Research Center, USA. The type of microarrays used includes fluorescent assays with indicated fluorescent dyes, and non-fluorescent assays (C: colorimetric/chemiluminescent assays; or E: assays with electric arrays) with indicated chromogenic dyes/chemiluminescent substrates. CSPD: chloro-5-substituted adamantyl-1,2-dioxetane phosphate; 6-FAM: 6-carboxyfluorescein; NS: not specified; PE: phycoerythrin; SG: Seramun Green; TAMRA: carboxytetramethylrhodamine; and TMB: 3,3′,5,5′-tetramethylbenzidine.

### 3.3. Merits of DNA Microarray Assay

Applications and potentials of DNA microarray technologies (e.g., DNA, cDNA and oligonucleotide microarray assays) for the detection and identification of microbial pathogens, such as antibiotic resistance genes, virulence factors and strain subtypes, have been discussed by comparison with other DNA-based methods, including PCR [224,225,226,227,228]. While PCR-based methods are normally limited to the analysis of a single or a small number of pathogens, microarray technology can analyze a significant number of pathogens simultaneously, and thus it has potential for use in basic research and industrial applications, such as food safety assessment. Gene expression profiling by DNA microarray assay has an advantage of examining the expression of large numbers of genes in a single experiment, and thus has been widely used to analyze food samples and materials. DNA microarray technologies have also been applied to monitor genetically modified food [225,229] and traditional Chinese medicine [230,231], and to evaluate drug safety [232]. Degenkolbe *et al.* discussed how quality control was examined for the procedures in DNA microarray assay, such as mRNA preparation, cDNA synthesis, fluorescent dye-labeling, hybridization/imaging and data analysis, using plant leaf tissue as a source of mRNA [233].

While DNA microarray assay has been considered to be effective and sensitive for assaying microbial spoilage of food, it is expensive and requires technical expertise. Therefore, several alternative methods were developed to explore cost-effective but still high-throughput assay systems. Böhme *et al.* developed an efficient method for bacterial identification based on detection of the 16S rRNA gene by flow-through hybridization on membranes, coupled to ligation detection reaction, which may provide an alternative to a DNA microarray assay for the rapid, accurate and cost-effective identification of bacterial species in order to assess food quality and safety [234]. Atanasova and Druzhinina discussed the Phenotype MicroArray, which tests cell respiration as a reporter system to characterize the metabolism of food spoilage pathogens, including conidial fungi [235]. However, the number of probes in these alternative assays is generally up to 100, and DNA microarray assays would be more useful when the number is over 100.

One of the merits of a DNA microarray assay is that it provides information about pathway-based intracellular signaling, which is important to evaluate the efficacy and mechanism of action of food materials. For example, a variety of signaling pathways have been identified by DNA microarray assay for traditional herbal medicine, such as traditional Chinese medicine (TCM) and traditional Japanese medicine (Kampo), which are associated with effects on cell functions and diseases, such as anti-adipogenesis, anti-atherosclerosis, anti-carcinogenesis, anti-inflammation, apoptosis, chemoprevention, circulation disorder and neuroprotection [231]. For example, the mitogen-activated protein kinase (MAPK) signaling pathway was shown to be associated with the apoptotic effect of Inchin-ko-to (Kampo) [236] and the anti-carcinogenic effect of Juzen-taiho-to (Kampo) [237], while TGF-β1/Smad and IGF-1 signaling pathways were associated with the inhibitory effect of Kangxianling (TCM) on renal fibrosis [238] and the immune response against viral infection induced by VI-28 (TCM) [239], respectively. The pathways associated with environmental estrogens, such as phytoestrogens, which are also important food materials, include a variety of signaling pathways related to apoptosis, carcinogenesis, cell growth/proliferation, differentiation/development and inflammation [240]. Therefore, the information about pathway-based intracellular signaling provided by DNA microarray assays will add variability and sensitivity to the assay system.

## 4. Conclusions

We have here summarized recent progress in fluorescence-based bioassays developed and applied for the detection and evaluation of food materials. A comprehensive list of fluorescent dyes used in recent bioassays includes those in biosensing, cell assay, energy transfer-based assay, probing, protein/immunological assay and microarray/biochip assay. Among these technologies, fluorescence-based microarrays/biochips, such as antibody/protein microarrays, bead/suspension arrays, capillary/sensor arrays, DNA microarrays/PCR-based arrays, glycan/lectin arrays, immunoassay/ELISA-based arrays, microfluidic chips and tissue arrays, have been developed and used widely for food safety and quality as well as the search for effective components. Applications of DNA microarray assay were discussed for important issues, such as allergy/poisoning/toxicity, contamination, efficacy/mechanism and quality control/safety, based on a comprehensive list of references showing these cases. The merits of DNA microarray assays were discussed by pointing to their advantages over other technologies in terms of features such as the sensitivity and efficiency, the number of probes to be analyzed rapidly and simultaneously, and the quality and quantity of information about pathway-based intracellular signaling in response to food materials.
